# Supplementary material for: Text mining in long-term care: Exploring the usefulness of artificial intelligence in a nursing home setting
Source: PLoS One. 2022 Aug 25;17(8):e0268281. doi: 10.1371/journal.pone.0268281 (PMC9409502; doi:10.1371/journal.pone.0268281)
Supplement: Attachment — Submitted filename: Response to Reviewers.docx [file pone.0268281.s001.docx]

**Author’s reply to reviewers’ comments:**

**Title:** Text mining in long-term care: exploring the usefulness of computer-aided methods of analysis

**Ref. No.** PONE-D-21-18516

The original reviewer’s comments are presented in *italics*. Our responses are presented in blue.

*Editor’s comments*

*1. Please ensure that your manuscript meets PLOS ONE's style requirements, including those for file naming.*

We have incorporated the styling guidelines and adjusted the manuscript accordingly.

*2. Please provide additional details regarding participant consent. In the ethics statement in the Methods and online submission information, please ensure that you have specified (1) whether consent was informed and (2) what type you obtained (for instance, written or verbal, and if verbal, how it was documented and witnessed). If your study included minors, state whether you obtained consent from parents or guardians. If the need for consent was waived by the ethics committee, please include this information."*

We have revised these statements to include the necessary information: information about the study was provided to all interviewers, residents, family members and caregivers in advance by an information letter. All participants provided written informed consent: residents with legal representatives gave informed consent themselves (as well as their legal representatives) before and during the conversations.

*3. Thank you for including your ethics statement: "Written ethical approval was provided (17-N-86 METC Zuyderland).".*

*a. Please amend your current ethics statement to include the full name of the ethics committee/institutional review board(s) that approved your specific study.*

The statement has been amended. The medical ethics committee of Zuyderland, the Netherlands, approved the study protocol (17-N-86) and concluded that the study was not subject to the Medical Research Involving Human Subjects Act.

*b. Once you have amended this/these statement(s) in the Methods section of the manuscript, please add the same text to the “Ethics Statement” field of the submission form (via “Edit Submission”).*

*For additional information about PLOS ONE ethical requirements for human subjects research, please refer to* [*http://journals.plos.org/plosone/s/submission-guidelines#loc-human-subjects-research*](http://journals.plos.org/plosone/s/submission-guidelines#loc-human-subjects-research)*..*

The statement has been amended here as well.

*Comments to the Author*

*Have the authors made all data underlying the findings in their manuscript fully available?*

*The PLOS Data policy requires authors to make all data underlying the findings described in their manuscript fully available without restriction, with rare exception (please refer to the Data Availability Statement in the manuscript PDF file). The data should be provided as part of the manuscript or its supporting information, or deposited to a public repository. For example, in addition to summary statistics, the data points behind means, medians and variance measures should be available. If there are restrictions on publicly sharing data—e.g. participant privacy or use of data from a third party—those must be specified.*

*Reviewer #1: No*

*Reviewer #2: Yes*

*Reviewer #3: No*

*Reviewer #4: Yes*

We have addressed this comment by sharing our sentiment analysis model and the source data of our figures (i.e. unigram frequencies (per group), bigram frequencies and sentiment of unigrams). These will become available on Github after publication. Moreover, all R and Python code will be become available on Github. Our interview data will not be publicly available due to the privacy of our participants. While certain privacy-related information was removed from the transcripts (e.g. names of participants, living addresses, room numbers and other personally identifiable information), the stories that our participants tell are often of a personal nature. Upon request, our interview data can be provided.

*5. Review Comments to the Author*

We would like to thank all 4 reviewers for their comments and suggestions for the paper. Three reviewers were very positive about our study and made concrete reflections and suggestions for improvement. We have responded to these in our rebuttal below. One reviewer was negative about the paper although there was no explanation why and suggestions for improvement were lacking.

*Reviewer #1: This study certainly does not represent both scientific and practical interest after complete revision according to my point of view the paper is not suitable for publication in this journal so it must be rejected*

We would like to thank the reviewer for his/her time. We’re sorry that we couldn’t satisfy your scientific and practical interest and we regret that you were unable to provide us any concrete comments regarding this decision.

*Reviewer #2: Very interesting research. An innovative approach to the subject. I believe that the work should continue. They raise a very important topic. Residents, family members and care professionals uttered respectively 285, 362 and 549 words per interview. Word frequency analysis showed that words that occurred most frequently in the interviews are often positive.*

We would like to thank the reviewer for his/her positive words regarding our manuscript. We believe that the manuscript improved because of the changes made upon all reviewers’ comments.

*Reviewer #3: I would like to thank the authors for their contribution. The study brings interesting insights, and it is also good to see that the authors are aware of possible limitations, which is certainly appreciated. Please consider my feedback below for the next version.*

*(1) The study presents interesting exploratory analysis, indeed. However, I see that there is a good opportunity for applying further empirical experiments. For example, data clustering or developing a classification model should be a valuable addition. In this way, the study would be properly positioned within the context text or data mining in general.*

The reviewer stresses and important point here: more analyses related to data clustering or classification would be a valuable addition. However, these are beyond the scope of this paper. The current manuscript has a different focus: to introduce the nursing/long-term care community, to the possibilities of AI, and more specifically, to text-mining. Hence, we believe that an additional analysis would make the article less comprehensible. Therefore, we believe this could better be addressed in a follow-up article. As a matter of fact, we are currently working on various deep learning analyses with BERT-based language models. In a new study, we’ll be discussing different classification analyses in detail and comparing them against a human baseline (e.g. the human form of analyzing qualitative data: open and axial coding). We are currently conducting a thematic analysis through multi-label classification, using labels (i.e. themes) that are related to quality of care.

*(2) Further, the study should also be positioned in line with the state-of-the-art methods of Natural Language Processing (NLP). The NLP research is currently dominated by the use of transformer models (e.g BERT). Further, there are specialized models (e.g. BioBERT), which might be quite applicable in the present work. In the recent years, earlier methods, such as bag-of-words, have been losing ground to transformers. Therefore, I believe that it is so important for the study to demonstrate that the authors are aware of such advances, which may be adopted as part of the future work, at least.*

As the reviewer states, a bag of words approach, such as the naïve bayes approach that we used in our study does not reach the same accuracy as modern transformer models. We initially chose a naïve bayes approach, because such a model is ‘easy’ to explain and it’s much easier to replicate than a deep learning approach. Replication of a transformer model is more difficult due to inference and training requiring many computational resources. We wanted to show that even a less advanced method could lead to interesting insights. Additionally, deep learning approaches may not always outperform traditional methods [3]. In our case, by using naïve bayes we achieved an accuracy of around 70%, whereas with a BERT-based deep learning approach we achieved 75%. As this does increase the reliability of our research, we’ve replaced the naïve bayes sentiment analysis with RobBERT which was fine-tuned for sentiment analysis using the interview data [2]. Often such models are fine-tuned on review data (e.g. book or movie reviews), however the language in such reviews is very different from the language used in our interview data. Because of this change, we’ve also mentioned the programming language Python in the methods section, as many deep learning packages depend on this programming language.

*(3) The introduction should refer to more recent work discussing or applying NLP in the healthcare context. For example:*

*https://doi.org/10.1109/JBHI.2021.3099755*

*http://dx.doi.org/10.5220/0010414508250832*

The manuscript now contains the first article the reviewer proposed: “An Accurate Deep Learning Model for Clinical Entity Recognition From Clinical Notes.” Additionally, we’ve included another recent study about natural language processing in a healthcare context: a study conducted in 2020 that discusses how a model such as RoBERTa can be used to assess the sentiment in tweets regarding the COVID 19 pandemic [1].

*(4) Furthermore, I recommend touching on the explainability aspect. Model explainability is currently a very active area of research in the healthcare domain in particular. I suggest that the authors may discuss that aspect as part of the future work as well.*

We’ve dedicated an additional paragraph to model explainability as part of the future work. In this section we discuss the explainability of both traditional machine learning approaches and deep learning approaches. We also shortly discuss the advantages and disadvantages of both these approaches regarding the explainability (page 16, line 315-321).

*(5) As a minor issue, I find the title a bit vague, the use of “computer-aided analysis” sounds too broad. I recommend using more focused terminology.*

We’ve decided to change the title to: ‘Text mining in long-term care: exploring the usefulness of artificial intelligence in a nursing home setting’.

*Reviewer #4: The objective of this research work is to evaluate the quality of care at a nursing home from the comments(narrative data) provided by the residents or their family members and care professionals as perceived by them. Text mining techniques were employed for analysis. Sentiment analysis was done to identify the emotions associated with these people from the words they uttered. The results of sentiment analysis could have been quantified as the authors rightly pointed out that it would be more significant to conclude the quality of care at the nursing home.*

Thank you for your review. We’ve made a number of improvements based on all reviewers’ feedback. The results of the sentiment analysis were quantified and visualized in Figure 5 of the manuscript.

References:

1. Azeemi AH, Waheed A. COVID-19 Tweets Analysis through Transformer Language Models. arXiv preprint arXiv:2103.00199. 2021 Feb 27. Available from <https://arxiv.org/pdf/2103.00199>
2. Wang, A., Singh, A., Michael, J., Hill, F., Levy, O., & Bowman, S. R. (2018). GLUE: A multi-task benchmark and analysis platform for natural language understanding. arXiv preprint arXiv:1804.07461. <https://arxiv.org/pdf/1804.07461.pdf>
3. Shwartz-Ziv, R., & Armon, A. (2022). Tabular data: Deep learning is not all you need. Information Fusion, 81, 84-90.
