# Supplementary material for: Text mining in long-term care: Exploring the usefulness of artificial intelligence in a nursing home setting
Source: PLoS One. 2022 Aug 25;17(8):e0268281. doi: 10.1371/journal.pone.0268281 (PMC9409502; doi:10.1371/journal.pone.0268281)
Supplement: Attachment — Submitted filename: Response to Reviewers.docx [file pone.0268281.s002.docx]

**Author’s reply to reviewers’ comments:**

**Title:** Text mining in long-term care: exploring the usefulness of artificial intelligence in a nursing home setting

**Ref. No.** PONE-D-21-18516

The original reviewer’s comments are presented in *italics*. Our responses are presented in blue.

*Journal requirements*

*Please review your reference list to ensure that it is complete and correct. If you have cited papers that have been retracted, please include the rationale for doing so in the manuscript text, or remove these references and replace them with relevant current references. Any changes to the reference list should be mentioned in the rebuttal letter that accompanies your revised manuscript. If you need to cite a retracted article, indicate the article’s retracted status in the References list and also include a citation and full reference for the retraction notice.*

The authors reviewed all references in the reference list: no retracted articles were detected. The authors did add additional references with respect to the added topic cluster analysis.

*6. Review Comments to the Author*

*Reviewer #2: I believe that the work fully deserves to be published in PLOS One. Original, innovative topic. I have no comments on the research work. Congratulations to the authors.*

We would like to thank the reviewer for his/her positive words regarding the originality and innovativeness of our work and corresponding manuscript.

*Reviewer #3: I thank the authors very much for their response, and for the amendments done.*

*However, I am afraid that I have to keep my initial recommendation that the manuscript would need further experiments to be considered for a journal publication, especially for a quite high-impact journal like PLOS ONE.*

*I find the argument about ”introducing the nursing care community to the possibilities of AI” not largely convincing. Applications of Text-Mining have already spanned a plethora of publications in the healthcare domain.*

We would like to thank the reviewer for his/her response. The authors decided to include a topic clustering analysis with the aim of discovering topics that were discussed in the interviews with residents, family members and care professionals. Keywords were extracted using POS tagging with a Dutch RobBERTa-like model. Nouns were selected to be representative of the topics existing in the interviews. Using word2vec, keywords were encoded as vectors. These vectors were transformed from cosine to Euclidian space and clustered using k-means. A value for k was calculated using the elbow method. A value of k = 12 was found to be optimal. Afterwards, each cluster was manually given a topic name based on the keywords that were included in the respective cluster. Based on this added analysis, the methods, results, and discussion were amended.
